# Delirium is frequently underdiagnosed among older hospitalised patients despite available information in hospital medical records

**DOI:** 10.1093/ageing/afae006

**Published:** 2024-02-10

**Authors:** Irit Titlestad, Kristoffer Haugarvoll, Stein-Erik H Solvang, Tone Merete Norekvål, Ragnhild E Skogseth, Ole A Andreassen, Dag Årsland, Bjørn Erik Neerland, Jan Erik Nordrehaug, Grethe S Tell, Lasse M Giil

**Affiliations:** Department of Clinical Medicine, University of Bergen, Bergen, Norway; Neuro-SysMed, Department of Internal Medicine, Haraldsplass Deaconess Hospital, Bergen, Norway; Neuro-SysMed, Department of Neurology, Haukeland University Hospital, Bergen, Norway; Neuro-SysMed, Department of Internal Medicine, Haraldsplass Deaconess Hospital, Bergen, Norway; Department of Heart Disease, Haukeland University Hospital, Bergen, Norway; Department of Clinical Science, University of Bergen, Bergen, Norway; Neuro-SysMed, Department of Internal Medicine, Haraldsplass Deaconess Hospital, Bergen, Norway; Department of Clinical Science, University of Bergen, Bergen, Norway; NORMENT, Division of Mental Health and Addiction, Oslo University Hospital & Institute of Clinical Medicine, University of Oslo, Oslo, Norway; Centre for Age-Related Medicine (SESAM), Stavanger University Hospital, Stavanger, Norway; Department of Old Age Psychiatry, Institute of Psychiatry, Psychology and Neuroscience, King’s College London, London, UK; Oslo Delirium Research Group, Department of Geriatric Medicine, Oslo University Hospital, Oslo, Norway; Department of Clinical Science, University of Bergen, Bergen, Norway; Department of Global Public Health and Primary Care, University of Bergen, Bergen, Norway; Neuro-SysMed, Department of Internal Medicine, Haraldsplass Deaconess Hospital, Bergen, Norway; Department of Clinical Science, University of Bergen, Bergen, Norway

**Keywords:** delirium, electronic medical records, discharge diagnosis, hospitalization, older people

## Abstract

**Background:**

In-hospital delirium is associated with adverse outcomes and is underdiagnosed, limiting research and clinical follow-up.

**Objective:**

To compare the incidence of in-hospital delirium determined by chart-based review of electronic medical records (D-CBR) with delirium discharge diagnoses (D-DD). Furthermore, to identify differences in symptoms, treatments and delirium triggers between D-CBR and D-DD.

**Method:**

The community-based cohort included 2,115 participants in the Hordaland Health Study born between 1925 and 1927. Between 2018 and 2022, we retrospectively reviewed hospital electronic medical records from baseline (1997–99) until death prior to 2023. D-DD and D-CBR were validated using *The Diagnostic and Statistical Manual of Mental Disorders, Fifth Edition,* criteria for delirium.

**Results:**

Of the 2,115 participants, 638 had in-hospital delirium. The incidence rate (IR) of D-CBR was 29.8 [95% confidence interval 28, 32] per 1,000 person-years, whereas the IR by D-DD was 3.4 [2.8, 4.2]. The IR ratio was 9.14 (*P* < 0.001). Patients who received pharmacological treatment for delirium (*n* = 121, odds ratio (OR) 3.4, [2.1, 5.4], *P* < 0.001), who were affected by acute memory impairment (*n* = 149, OR 2.8, [1.8, 4.5], *P* < 0.001), or change in perception (*n* = 137, OR 2.9, [1.8, 4.6] *P* < 0.001) had higher odds for D-DD. In contrast, post-operative cases (OR 0.2, [0.1, 0.4], *P* < 0.001) had lower odds for D-DD.

**Conclusion:**

Underdiagnosis of in-hospital delirium was a major issue in our study, especially in less severe delirium cases. Our findings emphasise the need for integrating systematic delirium diagnostics and documentation into hospital admission and discharge routines.

## Key Points

- In-hospital delirium is severely underdiagnosed, even though delirium symptoms are described in the medical records.
- A chart-based review reveals 9-fold additional delirium incidents than delirium discharge diagnosis alone.
- Patients with more severe delirium were more likely to receive a delirium discharge diagnosis in the discharge reports.
- Underdiagnosis and underreporting of delirium are likely to adversely affect clinical follow-up and delirium research.
- Implementation of systematic delirium diagnostics and documentation as a part of the hospitalisation routine care is needed.

## Introduction

Delirium is an acute neuropsychiatric syndrome that often manifests in conjunction with acute illnesses such as infections, fractures, surgeries and exposure to psychoactive drugs [1, 2]. In delirium, the patient’s cognitive ability is impaired in one or more cognitive domains resulting in, for example, fluctuating deficits in attention, arousal and/or perception. Patients with delirium frequently experience distressing hallucinations and delusions [2]. The risk of delirium depends on the severity of the acute illness and factors including age, co-morbidities, and asymptomatic or symptomatic neurodegenerative disease [3, 4]. A recent meta-analysis estimated the occurrence of in-hospital delirium at 23% [5]. However, estimates differ widely depending on age, frailty and prevalence of neurodegenerative disease in the study population [2]. Estimates also vary depending on the type of hospital ward and method of delirium detection [2, 6].

Patients with in-hospital delirium suffer poor health outcomes including prolonged hospital stays [1, 4, 7], 3-fold greater odds for mortality compared with patients without delirium [8] and risk for exacerbation of existing dementia [2, 3, 7, 9, 10]. Indeed, Richardson et al. reported that there is a considerable increase in risk of onset of dementia (odds ratio (OR) of 8.8) during one year of follow-up after an in-hospital delirium incident [11]. These findings are in line with a recent meta-analysis [12].

Delirium is potentially preventable and treatable and adversely affects prognosis [2], yet delirium is often undiagnosed [4, 13]. According to The National Institute for Health and Care Excellence, it is essential to ensure that the diagnosis is properly documented in the patient’s medical records [14]. Findings from a recent systematic review indicate that delirium incidents are insufficiently documented [13]: the description of the delirium event is often missing from the discharge report, and the diagnosis is often not coded in the hospital administrative system. The reported frequencies of descriptions of delirium in the discharge reports and coding of the diagnosis range widely between studies (0.1–64% and 1.5–49%, respectively) [13]. In a retrospective chart review of 110 patients with in-hospital delirium, 81% had the diagnosis described in the summary reports; however, only 31% received a formal delirium diagnosis [15]. Similarly, a large prospective study using the 4AT assessment tool and *The Diagnostic and Statistical Manual of Mental Disorders, Fifth Edition* (DSM-5) criteria found that only 29% of the delirium episodes were documented in discharge reports [4]. A recent population-based study has found substantially improved delirium coding rates in England and Scotland from 2012/2013 to 2019/2020. This improvement was observed across all age bands, especially among the oldest age groups (>80); however, the diagnosis rates remain underreported [16]. Simple tools such as the 4AT assessment test can detect in-hospital delirium [17] and is also a strong predictor of mortality [18].

A diagnosis of delirium status can be obtained from delirium discharge diagnoses (D-DD) that are stored in health registries without review of electronic medical records (EMRs). The discharge diagnoses are typically used for epidemiological and biobank studies that link baseline information to subsequent clinical events. The Norwegian Patient Registry (NPR) includes the International Classification of Disease (ICD) codes from all electronic hospital discharge diagnoses, but the validity of D-DD in the NPR remains unknown. Chart-based review methods can be used to identify delirium cases that were not reported in the discharge summaries [19].

The main aim of this study was to determine the incidence of in-hospital delirium as determined by a chart-based review of the electronic medical records (D-CBR) and compare this with the incidence based on D-DD. Furthermore, we aimed to identify clinical differences between D-DD and D-CBR cases in terms of delirium symptoms, targeted pharmacological treatment for delirium and the presumed trigger of delirium.

## Materials and methods

### Study participants and ethics

The participants in the current study were part of a large epidemiologic population-based study, the community-based Hordaland Health Study (HUSK). All the participants born 1925–27 who participated in HUSK in 1997–99 (age 70–74) were included (*N* = 3,273) [20]. See Supplementary Data for further information regarding the HUSK study and the current participants.

Our study was approved by The Regional Committee for Medical and Health Research Ethics (REK, project number 2016/2208). All participants signed informed consent at study start and gave permission for linkage to registries. However, the participants did not give active consent to allow review of EMRs. The Ethics committee therefore only approved linkage to hospital EMRs for deceased participants. Thus, the current analyses are based on a review of the EMRs of 2,115 participants who were deceased by 2022.

### EMRs review

We gathered information retrospectively on patients for whom data were available in 1997–99 (baseline) through the last record available from EMRs of Haukeland University Hospital and Haraldsplass Deaconess Hospital, the main hospitals in the region. The EMRs review included records from elective and acute admissions and outpatient clinics in the period 1997–2022. All entries from all hospital units were reviewed. A standardised protocol adapted for retrospective review of EMRs was developed based on diagnostic criteria established prior to data collection and modified during review of the first 150 cases. Data collected included the cause of admission, previous diagnoses, clinical information and whether the patients experienced delirium during hospitalisation. The ICD code for delirium, F05, in discharge summary reports was the proxy measure for a diagnosis.

### Identifying cases of delirium: screening and validation

Delirium was identified and validated based on entries in the EMRs that by themselves or cumulatively fulfilled the DSM-5 criteria for delirium [21]. The diagnostic process followed a chart-based method that is described in detail in the Supplementary Data. The delirium symptoms that occurred (e.g. impaired memory, confusion, disorientation, language difficulties, hallucinations and delusions) and the presumed precipitating cause were recorded (see Supplementary Data). Groups were defined based on their associations with previously reported risks of delirium [3, 14, 22–24]: infections, fractures, post-operative state and other medical conditions (see Supplementary Data for further details).

For the purpose of this study, we defined D-DD as cases with an ICD code of F05 in the EMRs discharge summary reports. D-CBR refers to delirium cases described in the EMRs, with a diagnosis based on the DSM-5 criteria, though not registered as a D-DD. Only the first delirium episode for each participant was included in the analysis.

### Statistics

We estimated incidence rates (IRs) per 1,000 person-years for D-DD and for D-CBR cases using the Stata command stptime, stratifying by baseline age and sex, and exploring IRs over 5-year intervals. By entering the person-years and number of cases for D-DD and D-CBR, we estimated the incidence rate ratio (IRR) with D-DD cases in the denominator using Fisher’s exact test to estimate the significance of any differences. Furthermore, we estimated the cumulative IRs of D-DD and D-CBR cases using the Stata command stcompet. The clinical variables affecting the odds of D-DD with D-CBR cases as the reference group were tested using logistic regression; ORs are reported. All analyses and figures were produced using StataCorp. 2023. *Stata Statistical Software: Release 18*. College Station, TX: StataCorp LLC.

## Results

### Study participants and follow-up

In total, 2,115 participants were included in our study (Table 1). Of these, 49.3% were men. Participants were selected based on year of birth (1925–27); thus, the age span was narrow, with almost all participants 71 (33.1%), 72 (32.6%) or 73 (31.4%) years old (2.9% were 70 and 0.05% were 74). Additional demographic details are provided in the Supplementary Data. Participants were followed until death. The median follow-up time was 12.4 years (interquartile range (IQR) 7.97), range 0.01–19.8 years. Of the 2,115 participants, 638 (30.2%) had at least one delirium episode during hospitalisation according to the review of EMRs, whereas D-DD was reported for only 84 participants (13.2%). All but two D-DD cases were also classified as D-CBR. The two exceptions were excluded as erroneous coding.

**Table 1:** Baseline demographics (*N* = 2,115). The Hordaland Health Study

			Number (percent)	
Age (years)				
	70		61 (2.9)	
	71		701 (33.1)	
	72		689 (32.6)	
	73		663 (31.4)	
	74		1 (0.05)	
Men			1,042 (49.3)	
All delirium cases^a^			638 (30.2)	
Delirium diagnosis (*n* = 638)				
	D-DD^b^		84 (13.2)	
	D-CBR^c^		554 (86.8)	

Abbreviations: D-DD, delirium discharge diagnoses; D-CBR, delirium by chart-based review of electronic medical records; EMRs, electronic medical records; DSM-5, *The Diagnostic and Statistical Manual of Mental Disorders, Fifth Edition*; ICD, International Classification of Disease; NPR, Norwegian Patient Registry.

^a^The median follow-up time was 12.4 years (IQR 7.97), with a minimum follow-up of 0.01 years and a maximum of 19.8 years (from 1997 to 2022). Only the first delirium episode of each participant was included in the analysis.

^b^Categorisation as D-DD was based on F05 coding by clinicians in the discharge summary reports and validation using DSM-5 diagnostic criteria. The ICD code is a proxy measure for what is recorded in the NPR.

^c^Categorisation as C-CBR was based on review of all EMRs in accordance with the DSM-5 diagnostic criteria, though the diagnosis was not registered as D-DD in the discharge summary report. Two of the D-DD cases were not classified as D-CBR as information in the EMRs did not meet the DSM-5 diagnostic criteria.

### Delirium incidence: D-DD versus D-CBR

**Figure 1:**
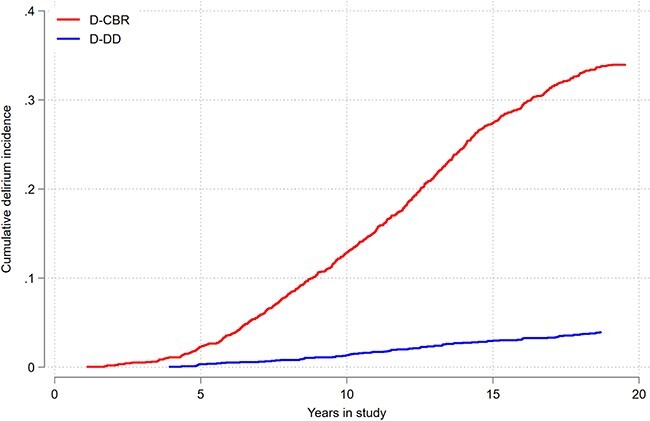
*The cumulative incidence of delirium according to source of diagnosis. The Hordaland Health Study. Delirium discharge diagnoses (D-DD) versus delirium identified by chart-based review of electronic medical records (D-CBR)*. The *y*-axis shows cumulative incidence from 0 to 0.4 (0–40%) and the *x*-axis displays years in study. The analysis included only the first delirium episode for each patient.

The IR of D-DD during hospitalisation was 3.4 [2.8, 4.2] per 1,000 person-years, whereas the IR of D-CBR was 29.8 [28, 32]. The IRR was 9.14 (*P* < 0.001) (Table 2). The cumulative incidences are plotted in Figure 1, showing a major increase in delirium incidence over time. Rates of D-DD and D-CBR both increased with age. The IRR decreased slightly with age, suggesting that D-DD is more frequently reported in older patients. Men had delirium more often than women, although the IRR was lower in men, suggesting that a lack of D-DD is more frequent in women. During the first 5 years of follow-up, there were only seven cases of D-DD. Ignoring the first 5 years of the study, the IRR was lowest at the end of the study period (15–20 years).

**Table 2:** Incidences of delirium per 1,000 person-years identified as delirium discharge diagnosis in the EMRs or through a systematic review of hospital EMRs. The Hordaland Health Study

			D-DD^a^			D-CBR^b^				
			Person years (events)	IR (95% CI)		Person years (events)	IR (95% CI)		IRR	*P* ^c^
All			24,683 (84)	3.4 [2.8, 4.2]		23,116 (638)	29.8 [28, 32]		9.14	<0.001
Age at baseline (years)										
	70–71		8,852 (23)	2.6 [1.7, 3.9]		8,314 (235)	28.2 [25, 32]		10.9	<0.001
	72		8,147 (28)	3.4 [2.4, 5.0]		7,638 (239)	31.3 [28, 36]		9.10	<0.001
	73–74		7,684 (33)	4.3 [3.1, 6.0]		7,164 (245)	34.2 [30, 39]		7.96	<0.001
Women			13,270 (33)	1.5 [1.0, 2.4]		12,370 (354)	27.6 [25, 31]		11.5	<0.001
Men			11,413 (51)	2.6 [1.8, 3.7]		10,746 (365)	32.2 [29, 36]		7.60	<0.001
Years follow-up										
	0–5		9,993 (7)	0.7 [0.3, 1.5]		9,954 (48)	4.8 [3.6, 6.4]		6.88	<0.001
	5–10		8,139 (21)	2.6 [1.7, 4.0]		7,733 (224)	29.0 [25, 33]		11.2	<0.001
	10–15		5,141 (35)	6.8 [4.9, 9.5]		4,414 (308)	69.8 [62, 78]		10.2	<0.001
	15–20		1,410 (21)	15.0 [9.7, 23]		1,045 (139)	137 [116, 162]		6.05	<0.001

Abbreviations: D-DD, delirium discharge diagnoses; D-CBR, delirium by chart-based review of electronic medical records; EMRs, electronic medical records; DSM-5, *The Diagnostic and Statistical Manual of Mental Disorders, Fifth Edition*; ICD, International Classification of Disease; NPR, Norwegian Patient Registry; IR, incidence rate; IRR, incidence rate ratios; *P*, *P* value.

^a^Categorisation as D-DD was based on F05 coding by clinicians in the discharge summary reports and validation using DSM-5 diagnostic criteria. The ICD code is a proxy measure for what is recorded in the NPR. Only the first delirium episode of each participant was included in the analysis.

^b^Categorisation as C-CBR was based on review of all EMRs in accordance with the DSM-5 diagnostic criteria, though the diagnosis was not registered as D-DD in the discharge summary report. Two of the D-DD cases were not classified as D-CBR as information in the EMRs did not meet the DSM-5 diagnostic criteria. Only the first delirium episode of each participant was included in the analysis.

^c^ *P* values were determined using Fisher’s exact test to compare IRs of D-DD and D-CBR per group (sex) and strata (age at baseline and years in study). Statistically significant *P* value < 0.05.

### Factors associated with the odds of receiving D-DD

Age at diagnosis was not associated with receiving a D-DD in the discharge report (OR 1.05 [0.99, 1.11], *P* = 0.115). Men received a D-DD more often than women (OR 1.58 [1.00, 2.52], *P* = 0.052). Patients classified by D-CBR with symptoms of memory impairment (OR 2.84 [1.78, 4.53], *P* < 0.001) or disturbances in perception (OR 2.88 [1.80, 4.60], *P* < 0.001) or who received pharmacological treatment for delirium (OR 3.36 [2.09, 5.40], *P* < 0.001) were more likely to receive a D-DD. Post-operative delirium episodes (OR 0.16, [0.06, 0.43], *P* < 0.001) were less likely to be reported at discharge with a D-DD. Delirium episodes that occurred in conjunction with infections, fractures and other medical conditions were not associated with the odds of receiving a D-DD (Table 3).

**Table 3:** Risk factors associated with receiving a delirium discharge diagnosis during a hospital stay. The Hordaland Health Study

	D-DD^a^ (*N* = 84)	D-CBR^b^ (*N* = 638)			
Symptoms and delirium-targeted treatment	Cum. events (%)^c^	Cum. events (%)^c^	OR^d^	95% CI	*P* ^e^
Pharmacological treatment for delirium	37 (44.1)	121 (19.0)	3.36	2.09, 5.40	<0.001^**^
Memory impairment	39 (46)	149 (23.3)	2.84	1.78, 4.53	<0.001^**^
Language impairment	11 (13.1)	69 (10.8)	1.24	0.63, 2.46	0.532
Perception disturbances	37 (44.1)	137 (21.5)	2.88	1.80, 4.60	<0.001^**^
					
Presumed underlying precipitating cause for delirium					
Post-operative state	4 (4.8)	155 (24.3)	0.16	0.06, 0.43	<0.001^**^
Fracture	13 (15.5)	109 (17.8)	0.88	0.48, 1.66	0.712
Infection	44 (52.4)	301 (47.2)	1.23	0.78, 1.94	0.370
Other medical conditions^f^	33 (39.3)	266 (41.7)	0.90	0.57, 1.44	0.674

Abbreviations: D-DD, delirium discharge diagnoses; D-CBR, delirium by chart-based review of electronic medical records; Cum. events, cumulative events; OR, odds ratio; CI, confidence interval; *P*, *P* value; EMRs, electronic medical records; DSM-5, *The Diagnostic and Statistical Manual of Mental Disorders, Fifth Edition*; ICD, International Classification of Disease; NPR, Norwegian Patient Registry.

^a^Categorisation as D-DD was based on F05 coding by clinicians in the discharge summary reports and validation using DSM-5 diagnostic criteria. The ICD code is a proxy measure for what is recorded in the NPR. Only the first delirium episode of each participant was included in the analysis.

^b^Categorisation as C-CBR was based on review of all EMRs in accordance with the DSM-5 diagnostic criteria, though the diagnosis was not registered as D-DD in the discharge summary report. Two of the D-DD cases were not classified as D-CBR as information in the EMRs did not meet the DSM-5 diagnostic criteria. Only the first delirium episode of each participant was included in the analysis.

^c^The cumulative number of persons with at least one delirium episode (either under D-DD or D-CBR) in the study period; percent of persons with at least one delirium episode during the study period relative to the study population is given in parentheses.

^d^Logistic regression was performed for each variable with D-DD as the outcome with D-CBR as the reference group; a higher OR indicates an increased probability of receiving a D-DD.

^e^Statistically significant *P* values are indicated as ^*^P < 0.05, ^**^P < 0.001.

^f^Medical conditions other than post-operative state, infections and fractures (e.g. myocardial infarction, hypoglycemia).

## Discussion

In this study, we compared the incidences of D-DD, which was a diagnosis of delirium reported in the discharge summary to incidences of delirium diagnosed by applying a chart-based method, D-CBR for a group of participants in the HUSK study. The chart-based method was based on careful review of available EMRs. We found that delirium was severely underdiagnosed in hospital discharge summary reports: our review of EMRs revealed IRs that were nine times higher than those reported at discharge with an ICD code of F05 (D-DD). Such under-reporting adversely affects both patient care and delirium research. Patients with a D-DD were more likely to be those who suffered perceptual disturbances or memory impairment or those for whom pharmacological treatment targeting delirium was provided, although even these cases were underdiagnosed. Patients with post-operative delirium were unlikely to receive a D-DD.

Our study demonstrated that despite information in EMRs, the number of in-hospital delirium cases was severely underestimated. Our findings are consistent with those of Ibitoye et al. who showed that delirium is under-coded and under-documented in discharge summary reports even when the diagnosis is detected [13]. However, significant improvements in the formal documentation of delirium have been observed in recent years [16]. Moreover, our findings demonstrated that underdiagnosis of delirium cases was more severe than in previous studies [4, 15].

Underdiagnosis of delirium may be due to the busy clinical setting of a hospital ward [25], the fluctuating course of delirium and the rotation of physicians, nurses and wards during the hospital stay [26]. Changes in staff due to schedule rotations or due to transfer of patients between wards may mean that healthcare staff do not necessarily observe acute alterations in the patient’s cognitive function due to a lack of familiarity with the pre-delirium status. Moreover, insufficient knowledge among healthcare staff regarding diagnosis of delirium may mean that symptoms of delirium are interpreted and described as confusion [2, 27], and it is challenging for healthcare staff to differentiate delirium from dementia [10]. Furthermore, our experience during review of EMRs was that delirium descriptions were mainly reported in nurses’ notes, often during evening and night shifts, and were not part of a structured delirium assessment. These notes may not consistently be reviewed by physicians. In the current study, only physicians were authorised to register a D-DD. Nurses commonly observe patients significantly more often than physicians and other healthcare staff. Thus, we speculate that coding of delirium would increase if delirium screening were integrated into nursing responsibilities. Systematic screening of delirium is important for prognosis [18] and may positively affect patient outcomes [28].

Our analysis showed that the IRRs were the lowest at the beginning (years 0–5) and at the end of the study (years 15–20). If the first 5 years are ignored, this suggests that D-DD became slightly more frequent over time. A possible explanation could be increased awareness among clinicians as in-hospital delirium research has demonstrated delirium-related short- and long-term complications and associations of delirium with mortality [8, 29, 30]. The extent of under-coding varies across healthcare systems, whereas in some systems, detection and formal diagnosing of delirium have improved over the years [16, 18]. We suggest that future studies should analyse factors that may impact delirium detection and D-DD rates, including educational factors, diagnostic tools and national and institutional guidelines. Furthermore, several studies have demonstrated the importance of preventative measures and of treatment for delirium [2, 31, 32]. In support of the possibility of increased awareness, an increase in delirium documentation was demonstrated in recent systematic reviews [13, 16]. It is also possible that clinicians may think about delirium more often as their patients age. As the IRR of D-CBR to D-DD was 6.05 at the end of our study period, underdiagnosis remained high. We observed a considerable increase in delirium incidence over the years of the study, in agreement with previous work that showed that the risk for delirium is positively associated with age [3, 4].

We observed significant differences between the numbers of D-DD and D-CBR cases. Disturbed perception, memory impairment and pharmacological treatment for delirium increased the odds of D-DD. This suggests that delirium is more often formally diagnosed when patient care due to delirium is more resource-consuming for health providers. These symptoms would typically be seen among patients with hyperactive delirium [33, 34].

Furthermore, our findings show that post-operative delirium was unlikely to be reported as a D-DD. Post-operative delirium is a common complication among older patients [24, 35]. Underdiagnosis of post-operative delirium may be because delirium is seen as a ‘normal’ condition in surgical wards or may be due to inadequate awareness and knowledge of the complexities of treating older patients [2, 27]. The detrimental effects of delirium may also be underestimated compared with other post-operative complications [36]. Our findings suggest that education of healthcare staff may improve formal reporting of delirium at discharge.

Underdiagnosis of delirium is a missed opportunity for implementing preventative measures on subsequent admissions, as patients who suffer one delirium incident are at risk of future episodes of delirium [37, 38]. Importantly, delirium can be an early symptom of undiagnosed neurodegenerative disease and is a major risk factor for onset of dementia and worsening of cognition [1, 3, 7, 9, 11]. Therefore, it is essential that delirium incidents be formally reported in discharge summaries so that cognitive screening can be performed after acute symptoms recede [9].

A hip fracture register report revealed that delirium screening was performed among over 80% of the patients hospitalised for hip fracture using the delirium assessment tool, 4AT; 25% of these patients were identified as having possible delirium [35]. These findings may suggest that registries that include delirium as an important key performance indicator may increase the diagnosing and detecting rate of delirium. The quality of registry data is crucial for research [39], and our study suggests that the use of registry data for delirium studies will lead to low statistical power and selection bias towards certain groups, unless the registries use methods which show effective delirium ascertainment [35]. To identify genetic and acquired traits correlated with susceptibility to delirium episodes, longitudinal studies of community-dwelling persons or the general population will be needed.

Our study has several strengths and limitations. To our knowledge, this is the largest cohort analysed in an assessment of the documentation of delirium among older hospitalised patients involving a complete review of EMRs. The study population had a narrow age range and thus the age-variation in the incidence of delirium was controlled for by the design. We used the DSM-5 criteria for establishing a diagnosis after a systematic review of available hospital EMRs and detected a substantial number of nonregistered delirium incidents. We used the DSM-5 to define delirium; other coding systems, such as ICD-10 and DSM-4, could have been used. Studies on cardiac surgery patients have estimated that the ICD-10 definition underestimates the presence of delirium compared with DSM-4 [40] but only by a few percent. Compared with DSM-4, DSM-5 is restrictive [41, 42] and it is thus not likely to be more permissive than ICD-10. It should be noted that the authority for a healthcare professional to report a diagnostic code for delirium varies from one healthcare system to another. Thus, coding practices in different countries may lead to additional variability in case definitions.

The response rate in the first HUSK study (1992–93) was 0.73, and in the second HUSK study in 1997–99 (the baseline of the current study) **0.77.** From the initially invited participants, who were selected by year of birth and residency within the selected municipalities, the participation rate in the 1997–99 HUSK study was 0.51 [20]. There is likely some bias towards healthier and less cognitively impaired participants with fewer risk factors for delirium at baseline. This means that the true IR of in-hospital delirium is likely higher in this community than reported here. Furthermore, hypoactive delirium, which is characterised by increased passivity, is challenging to identify and was likely significantly under-detected in our study, as it was in previous work [33]. Moreover, the retrospective design led to non-standardised entries in records. This study represents an interim analysis of a long-term project with only partial linkage to the baseline data (age and sex). Thus, variation in the underdiagnosis of delirium will be a topic of future studies.

In conclusion, we found that delirium affected approximately one of three community-dwelling, hospitalised patients over the course of 20 years beginning when the participants were aged ~70 years. Delirium was rarely registered as a discharge diagnosis, even though delirium symptoms and treatment were often described in EMRs. Thus, underdiagnosis of delirium remains an issue for researchers who are following this growing segment of the population. Our findings suggest that delirium is formally reported more frequently in more severe cases and emphasise the need to integrate systematic delirium diagnostics and its documentation as part of the admission, care and discharge routines.

## Supplementary Material

aa-23-0771-File002_afae006

## Data Dvailability

Due to ethical restrictions, the full data set is available to the reader upon request only. Proposals should be directed to the Project Manager at lassegiil@gmail.com and to gain access, data requestors will need to sign a data access agreement.
