# Supplementary material for: Delirium is frequently underdiagnosed among older hospitalised patients despite available information in hospital medical records
Source: Age Ageing. 2024 Feb 10;53(2):afae006. doi: 10.1093/ageing/afae006 (PMC10859244; doi:10.1093/ageing/afae006)
Supplement: aa-23-0771-File002_afae006 [file aa-23-0771-file002_afae006.docx]

**SUPPLEMENTARY DATA**

Delirium is frequently underdiagnosed among older hospitalized patients despite available information in hospital medical records

**CONTENTS LIST**

[1.0 SUPPLEMENTARY METHODS 2](#_Toc151376762)

[1.1 Study participants - The community-based Hordaland Health Study 2](#_Toc151376763)

[1.2 The diagnostic process of delirium by a chart-based review 3](#_Toc151376764)

[1.2.1 *The Diagnostic and Statistical Manual of Mental Disorders, Fifth Edition* criteria 3](#_Toc151376765)

[1.2.2 Definition of the presumed precipitating cause for delirium by groups 4](#_Toc151376766)

[1.2.3 Screening and identification procedure of delirium in the hospital electronic medical records 5](#_Toc151376767)

[2.0 REFERENCES 8](#_Toc151376768)

# **1.0 SUPPLEMENTARY METHODS**

## **1.1 Study participants - The community-based Hordaland Health Study**

The community-based Hordaland Health Study (HUSK) was conducted in Hordaland County, Western Norway, as cooperation between the University of Bergen, the Norwegian Health Screening Service, and the Municipal Health Service in Hordaland [1, 2]. During 1992-93, all persons born 1925-1927 and living in Bergen and three nearby suburban municipalities selected for the study were invited to participate. The first data gathering was conducted in 1992-1993 (HUSK 1) with a response rate of 0.73. Subsequently, in 1997-1999, the participants from HUSK 1 were invited to participate in a follow-up study (HUSK 2). In total, 3341 participants from HUSK 1 agreed to participate in HUSK 2, a response rate of **0.77** [2]. In the current study, we included all the participants from HUSK 2 who also donated blood samples (N=3273). The current study was only permitted to collect data from deceased participants (N=2115).

As the baseline data from HUSK 1 and HUSK 2 are unavailable at this stage of the project, we lack information regarding the participant demographics except for age and sex. However, data from a cross-sectional study [3], which included 2174 of the 3273 participants who were selected for cognitive testing based on their place of residence, reported relevant demographic information. Data included education level, smoking status, body mass index (BMI), and occurrence of specific diseases (e.g., diabetes, hypertension, depression). The education level was reported in five categories: 7% of the participants went to primary school for less than seven years and 32% for seven to ten years; 30% went to high school for one to two years, and 12% for three years. In total, 19% had studied at a college or university. Moreover, during data gathering in 1997-1999, 18% were smokers, and the mean BMI of the participants was 26.1 (standard deviation 3.9). The point prevalence of diabetes was 7%, 33% had hypertension, and almost 5% had suffered a stroke [3]. The baseline assessment (in 1997-1999) included a general physical examination, and participants responded to self-reported questionnaires to systematically assess lifestyle, dietary and smoking habits, medication usage, alcohol consumption, and risk factors for cardiovascular disease and diabetes mellitus [1, 2]. Additionally, the majority of participants submitted to a limited neuropsychological test battery [4].

## **1.2 The diagnostic process of delirium by a chart-based review**

The research team that gathered the data consisted of one physician and three trained study nurses with clinical experience. The identification and validation of delirium were based on information available in electronic medical records (EMRs), also known as a chart-based method [5]. *The* Diagnostic and Statistical Manual of Mental Disorders, Fifth Edition (DSM-5) criteria were applied to detect and define cases of delirium [6].

### **1.2.1 *The* Diagnostic and Statistical Manual of Mental Disorders, Fifth Edition criteria**

We defined the diagnosis of delirium when all DSM-5 criteria for diagnosis of delirium were met. The DSM-5 diagnostic criteria for delirium are the following [6]:

1. Disturbance in attention and awareness.
2. The disturbance develops over a short period of time, represents an acute change from baseline attention and awareness, and tends to fluctuate in severity during the day.
3. An additional disturbance in cognition (e.g., impaired memory, disorientation, language difficulties, impaired perception such as hallucinations and delusions).
4. The disturbances in Criteria A and C are not better explained by a pre-existing, established, or evolving neurocognitive disorder and do not occur in the context of a severely reduced level of arousal such as a coma.
5. There is evidence from the history, physical examination, or laboratory findings that the disturbance is a direct physiological consequence of another medical condition, substance intoxication or withdrawal, or exposure to a toxin, or is due to multiple etiologies.

### **1.2.2 Definition of the presumed precipitating cause for delirium by groups**

The precipitating causes of delirium were determined by review of EMRs. The potential causes were grouped as follows:

- **Infections,** all viral and bacterial infectious conditions.
- **Fractures**, all types of fractures. In cases where delirium occurred in conjunction with fractures that required surgical intervention, both fracture and surgery were registered as presumed causes.
- **Post-operative state** refers to the period following all surgical procedures.
- **Other medical conditions** refer to any acute medical conditions that cannot be classified into the categories above. These conditions included, for example, hyperglycemia and myocardial infarction.

In cases with more than one presumed precipitating cause for delirium (e.g., a postoperative state and an infection) both were included.

### **1.2.3 Screening and identification procedure of delirium in the hospital electronic medical records**

To identify participant diagnosed with delirium in discharge reports (D-DD), the research team started by reviewing the discharge summary reports and identifying whether they contained delirium discharge diagnoses (using the International Classification of Diseases coding system, code F05) or a description of symptoms in the text indicating delirium. Regardless of whether the discharge summary reports contained F05 and or a description of delirium in the text, the research team reviewed the admission reports (written by nurses and physicians) for information regarding the habitual cognitive function and mental status at admission. Information from relatives regarding cognitive function and recent acute changes in cognition was considered reliable. Details regarding the patient's mental status from the admission notes were necessary to identify acute changes in cognition during hospitalization.

Following the review of the discharge and admission reports, the research team reviewed all the EMRs registered during the hospitalization period (documented by physicians, nurses, physiotherapists, occupational therapists, and referring physicians). The research team searched for descriptions that indicated a change in cognition from the time of admission including indications of fluctuations in mental state, for example, cases where on admission, the patient was described as oriented for time and place, and later in the night shift, as disoriented and agitated. In cases of delirium, delirium symptoms and fluctuation courses were most often documented in the medical records written by nurses from the evening and night shifts. These records usually contained descriptions of delirium symptoms such as new onset of language or memory difficulties, disorientation, agitation, restlessness, inappropriate behavior, delusions, or hallucinations. In cases of suspected delirium in patients with dementia or pre-existing cognitive impairment, we looked for a fluctuated worsening of cognitive function. The information source regarding pre-existing dementia or chronic cognitive impairment was often found in the records from the geriatric outpatient clinic, admission records, or notes from the referring physician (usually under the "previous diagnosis" section).

The presumed triggering cause of delirium was determined using the information documented in the EMRs prior to and during delirium. For example, when the delirium symptoms appeared shortly before, simultaneously, or briefly after an acute medical or surgical condition (e.g., infection, fracture, operation, and myocardial infarction), the current acute medical/surgical condition was identified as the presumed triggering cause. We used all available information from the reports in the EMRs including notes by nurses, physicians, occupational therapists, and physiotherapists. In most cases, we also used information from laboratory results to identify and validate several acute medical conditions. The laboratory results were screened from a few days before occurrence of the delirium symptoms until the date of the delirium episode. In cases of doubt regarding the presumed cause, a physician reviewed the case, and the research team aimed to reach a consensus.

All EMRs were screened to assess whether the DSM-5 criteria were fulfilled including in cases where the patients received delirium discharge diagnoses. To determine whether the DSM-5 criteria (A-E) for delirium were fulfilled, the research team was required to find evidence for the presence of disturbance in attention and awareness (criterion A). For example, if the patient was described as confused, somnolent, or falling asleep during a conversation or physical examination, criterion A was met. Further, to meet the criteria for diagnosis of delirium it was required to find evidence that the acute cognitive impairment symptoms mentioned above (criteria C) appeared in conjunction with a medical or surgical condition (criteria E), were fluctuating (criteria B), and were not due to a pre-existing neurocognitive disorder (criteria D).

The research group performed a similar validation for patients who received a delirium discharge diagnosis (F05) in the discharge summary reports to ensure that the diagnosis met the DSM-5 criteria.

Mainly hyperactive delirium was identified and validated, whereas hypoactive delirium was challenging to detect based on a retrospective review of EMRs. Additionally, the pharmacological treatment for delirium was reported only when it was documented that it was administrated due to delirium or delirium-characterized behavior (e.g., restlessness, inappropriate and agitated behavior). Moreover, the current study did not include data regarding repeated delirium episodes from the same admission or subsequent admissions.

The strategy for reviewing EMRs and identifying delirium cases using the DSM-5 criteria was developed by trial and error during the first 150 cases. The first 150 cases were reviewed a second time (by the same abstractor) for quality control purposes. In cases where there was uncertainty regarding delirium occurrences, two or three clinical researchers discussed the case, and the case definition was defined by consensus. This was done to enhance the confidence of the researchers in detecting relevant phrases, defining cases by gaining experience, and receiving feedback.

# **2.0 REFERENCES**

1. The Hordaland Health Studies. HUSK-English - The Hordaland Health Studies Bergen, Norway n.d [09.02.2023]; Available from: <https://husk-en.w.uib.no/>.

2. Refsum H, Nurk E, Smith AD. et al. The Hordaland Homocysteine Study: A Community-Based Study of Homocysteine, Its Determinants, and Associations with Disease. *J Nutr* 2006; 136: 1731-40. DOI: 10.1093/jn/136.6.1731s

3. Solvang SH, Nordrehaug JE, Tell GS. et al. The kynurenine pathway and cognitive performance in community-dwelling older adults. The Hordaland Health Study. *Brain Behav Immun* 2019; 75: 155-62. DOI: 10.1016/j.bbi.2018.10.003

4. Skogen JC, Øverland S, Smith AD, Mykletun A, Stewart R. The impact of early life factors on cognitive function in old age: The Hordaland Health Study (HUSK). *BMC Psychol* 2013; 1: 16.

5. Inouye SK, Leo-Summers L, Zhang Y, Bogardus Jr ST, Leslie DL, Agostini JV. A Chart-Based Method for Identification of Delirium: Validation Compared with Interviewer Ratings Using the Confusion Assessment Method. *J Am Geriatr Soc.* 2005; 53: 312-8. DOI: 10.1111/j.1532-5415.2005.53120.x

6. American Psychiatric Association. *Diagnostic and statistical manual of mental disorders*: *DSM-5*. 5th ed. Washington D.C: American Psychiatric Association; 2013.
